# How chromatic cues can guide human eye growth to achieve good focus

**DOI:** 10.1167/jov.21.5.11

**Published:** 2021-05-13

**Authors:** Timothy J. Gawne, Rafael Grytz, Thomas T. Norton

**Affiliations:** 1 Department of Optometry and Vision Science, University of Alabama at Birmingham, Birmingham, AL, USA; 2 Department of Ophthalmology and Visual Sciences, University of Alabama at Birmingham, Birmingham, AL, USA; 3 Department of Optometry and Vision Science, University of Alabama at Birmingham, Birmingham, AL, USA

**Keywords:** emmetropization, myopia, longitudinal chromatic aberration

## Abstract

The postnatal growing eye uses visual cues to actively control its own axial elongation to achieve and maintain sharp focus, a process termed *emmetropization*. The primary visual cue may be the difference in image sharpness as sensed by the arrays of short- and long-wavelength sensitive cone photoreceptors caused by longitudinal chromatic aberration: Shorter wavelengths focus in front of longer wavelengths. However, the sparse distribution of short-wavelength sensitive cones across the retina suggests that they do not have sufficient spatial sampling resolution for this task. Here, we show that the spacing of the short-wavelength sensitive cones in humans is sufficient for them, in conjunction with the longer wavelength cones, to use chromatic signals to detect defocus and guide emmetropization. We hypothesize that the retinal spacing of the short-wavelength sensitive cones in many mammalian species is an evolutionarily ancient adaption that allows the efficient use of chromatic cues in emmetropization.

## Introduction

It has been well established that a feedback mechanism in the postnatal growing eye in humans and other species uses visual (optical) cues to evaluate the focus of the eye and to regulate the rate of axial elongation to achieve, and then actively maintain, sharp focus on the retina through childhood and into at least early adulthood (Norton, 1999; Troilo et al., 2019). If the eye is too short for its optics, it is termed *hyperopic*, and elongation is accelerated; if the eye is too long, it is termed *myopic*, and elongation is retarded until the increasing focal length of the growing eye moves the focal plane back onto the retina. This process is termed *emmetropization*. Maintaining emmetropia is critically important for achieving good acuity. In a 24-mm human eye, a mismatch of as little as 175 µm between the location of the focal plane, where images are in focus, and the retina, where images are detected, causes a 0.5-diopter (D) refractive error which reduces visual acuity from 20/20 to about 20/30 (US units; these levels of visual acuity would be represented as from 6/6 to 6/9.5 in most countries using the metric system) (Atchison, Jones, Schmid, Pritchard, Pope, Strugnell, & Riley, 2004).

In contrast to perceptual visual acuity, the emmetropization mechanism does not require the central fovea, where acuity is highest, but appears to depend on the peripheral retina (Smith et al., 2007). Indeed, emmetropization is primarily a local process that does not require the rest of the brain. A region of retina evaluates visual cues over time, and then a signaling cascade through the retinal pigment epithelium and choroid causes the underlying sclera to become either more or less extensible, thus adjusting the growth of the eye to maintain the foveal retina at the focal plane (Troilo et al., 2019). The localized retinal control has been demonstrated in chicks (Wallman, Gottlieb, Rajaram, & Fugate-Wentzek, 1987), tree shrews (Norton & Siegwart, 1991), and non-human primates (Smith, Huang, Hung, Blasdel, Humbird, & Bockhorst, 2009) with the use of diffusers or lenses that cover only part of the visual field, where only the part of the sclera adjacent to the retina receiving degraded images elongates. However, the precise nature of the visual cues used by the retina to guide emmetropization has remained unclear.

Unlike the lenses in cameras or microscopes, vertebrate eyes exhibit substantial longitudinal chromatic aberration (LCA): The shorter wavelengths of light focus approximately 2 D closer to the front of the eye than the longer wavelengths (Mandelman & Sivak, 1983). LCA is conserved across species and, in humans, is highly consistent across individuals (Thibos, Ye, Zhang, & Bradley, 1992). Thus, LCA could be a robust cue used by the emmetropization mechanism: If the array of short-wavelength sensitive cones detects a relatively sharper image than does the array of longer wavelength sensitive cone photoreceptors, this can indicate hyperopic defocus; the inverse condition would indicate myopic defocus (Rohrer, Schaeffel, & Zrenner, 1992; Rucker, Eskew, & Taylor, 2020; Rucker & Kruger, 2006; Wildsoet, Howland, Falconer, & Dick, 1993).

In non-color-blind humans, there are two longer wavelength sensitive cones: the middle-wavelength sensitive (MWS) and long-wavelength sensitive (LWS) cones. However, the peak wavelength sensitivities of human MWS cones (∼530 nm) and LWS cones (∼560 nm) are very close together and relatively long, and there is considerable evidence that emmetropization pools the signals from the MWS and LWS cones (Gawne & Norton, 2020). Here, we refer to them jointly as the MLWS cones. The MLWS cones are abundant across the surface of the retina; however, the short-wavelength sensitive (SWS) cones (peak sensitivity ∼425 nm) are relatively sparse across the retinal surface and incapable of resolving the high spatial frequencies of human vision (Calkins, 2001; Rohrer et al., 1992). To the extent that this is necessary for the SWS cone array to signal defocus, it would suggest that it would be difficult for emmetropization to use LCA as a cue for defocus. However, experiments using animals where LCA-based cues are not available have typically found that the emmetropization mechanism is not able to achieve or maintain good focus, suggesting that LCA-based cues provide essential information for emmetropization (Gawne, Ward, & Norton, 2017; Gawne, Ward, & Norton, 2018; Smith, Hung, Arumugam, Holden, Neitz, & Neitz, 2015). Some studies have suggested that animals can emmetropize in narrow-band light where chromatic cues are unavailable, but this could have been purely accidental; that is, with no feedback emmetropization could sometimes move in the correct direction by chance (for discussion, see Gawne et al., 2018; Smith et al., 2015). But how could LCA be used as a cue for emmetropization if the short wavelength cones have such poor spatial resolution?

The answer may be that the emmetropization mechanism uses lower spatial frequency information to guide eye growth. This is important, because a guidance system that depends on high-spatial frequency information would not work when images are out of focus because high-spatial frequency information is greatly reduced in defocused images. We have previously developed an optical model of emmetropization for tree shrews in which the retinal array of SWS cones generates a sampled image of the visual scene, and the retinal array of longer wavelength-sensitive cones generates a second, separate sampled image (Gawne & Norton, 2020). Here we have modified this model for human parameters and simulated the relative activation of short-wavelength and longer wavelength cones as a function of optical defocus for 25 real-world hyperspectral images.

## Methods

**Figure 1.**
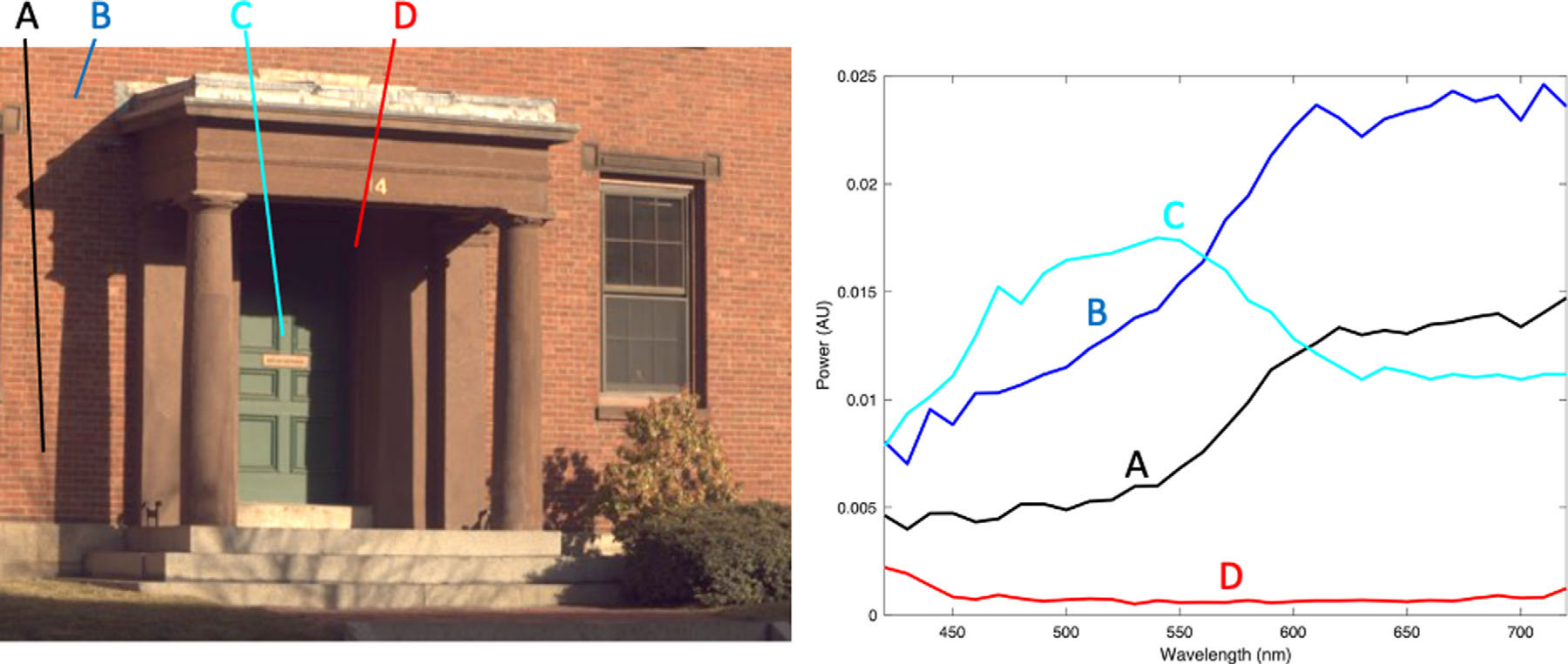
Example hyperspectral image from a publicly available database (Chakrabarty & Zickler, 2011). The picture on the left is the red–green–blue representation of the hyperspectral image, in which each pixel has the full spectrum spanning the range of visible light. Spectra from four image pixels are shown on the right.

There is an important distinction between the human perception of colors and the use of wavelength to guide eye growth. Because perceptual color vision is based on the relative activations of red, green, and blue cones, with just three monochromatic light sources one can cover approximately 80% of the human color gamut (Song, Li, & Liu, 2018). In practice, finite spectral bandwidth limits the color gamut of contemporary computer monitors and flatscreen televisions to less than this, but nonetheless one can generate such a broad range of perceptual colors with mixtures of red, green, and blue that much of our video technology is based on this. However, because the focal plane of light varies continuously with wavelength, for studies of emmetropization we must consider the entire visible spectrum (Autrusseau, Thibos, & Shevell, 2011). Therefore, in the present study, we used a set of 25 *hyperspectral* images that were obtained from a publicly available database (Chakrabarty & Zickler, 2011). Hyperspectral images differ from the images of a standard digital camera in that, rather than each pixel containing only three spectral values (red, green, and blue), each pixel contains intensity values at multiple wavelengths spanning the entire visible spectrum (Lu & Fei, 2014). An example of one of the hyperspectral images that we used is given in Figure 1. For each hyperspectral image, we calculated the effective images as sensed separately by the SWS and MLWS cone arrays as a function of optical defocus.

The present model extends to humans and to full-spectrum (hyperspectral) images a model of emmetropization that was developed in dichromatic tree shrews using monochromatic images (Gawne & Norton, 2020). This model assumes that emmetropization is guided by differences in the focus of shorter wavelengths, detected by the array of SWS cones, and the focus of longer wavelengths by the array of pooled MWS and LWS cones (MLWS). Greater image clarity detected by the SWS cone array, relative to the MLWS cone array, produces a drive for the eye to elongate. Greater relative image clarity as detected by the MLWS cone array produces a drive to slow eye growth. For each of 25 hyperspectral images, we calculated, for several levels of myopic (minus) and hyperopic (plus) defocus, the pattern of activation of the arrays of S and ML cone photoreceptors. More specifically, for each image and level of defocus, we derived a separate functional image.

We assumed that the optical power of the human eye is 60 D (Keating, 2002), which results in a focal length of 16.667 mm (1000 mm × 1/60). A power of 59 D would result in a focal length of 16.949 mm, so 1 D of defocus should correspond to an axial distance of approximately 282 µm. One degree of visual angle (≈ 1/57 radian) times the focal length translates to a distance across the retina of about 291 µm. We used a set of publicly available hyperspectral images (http://vision.seas.harvard.edu/hyperspec/) (Chakrabarty & Zickler, 2011). The total size of the download is large, at around 8.7 gigabytes, and the authors ask that readers wanting copies of these images email them for the download link (ayanc[at]eecs[dot]harvard[dot]edu).

Each image spans 1392 pixels horizontally and 1040 pixels vertically. Each individual pixel contains spectral data from 31 different wavelength bands, from 420 nm to 720 nm in 10-nm increments. We assume that each spatial pixel in the image maps is 4 µm across the surface of the retina; therefore, an image 1392 pixels wide would span about 5568 µm across the retinal surface and correspond to about 19.1 degrees of visual angle. Because real-world images tend to have similar statistics at different scales (Field, 1987; Ruderman, 1994) and because any given image can be arbitrarily made to occupy a smaller or larger field of view just by moving closer or farther away, we made no attempt to match the real-world distance from the visual scene to the camera.

We note that we have assumed that our images were all at optical infinity. For outdoor scenes, most of the visual world is at approximately the same constant level of defocus (Flitcroft, 2012), so this seems a reasonable assumption. Indoor scenes, however, have a wide variety of distances to different objects, and, even though the emmetropization system mostly is driven by the most distant objects (Troilo et al., 2019), it has been proposed that this lack of “dioptric flatness” in indoor scenes could interfere with emmetropization (Flitcroft, 2012), a possibility that we do not consider here but which should be explored.

We excluded two images as outliers because they were nearly devoid of spatial texture. Because hyperspectral image capture is slow, moving objects such as trees in wind can cause significant spatial and spectral artifacts. Therefore, we used only a subset of 25 of the images that did not have any motion artifact (as determined by the authors of the database; a list of the specific images used is shown in Supplementary Figure S1). At each wavelength, we divided by the sensitivity function of the hyperspectral camera (file calib.txt in the database) to yield equal sensitivity per spectral frequency band.

Normal human pupil size can vary from 2 to 4 mm in bright light and from 4 to 8 mm in dim light (Spector, 1990). Here, we assumed an intermediate pupil diameter of 4 mm. We calculated the amount of LCA at a given wavelength by the following previously published formula (Thibos et al., 1992):

$$\begin{array}{c} Rx=p-q/\left(lambda-c\right)\\ p=1.68524\\ q=0.63346\\ c=0.21410 \end{array}$$

where *lambda* is the wavelength (in µm; divide by 1000 to convert from nm), and *Rx* is the LCA (in diopters).

This published formula sets zero LCA at 600 m, but we added 0.53 D to *Rx* to re-center the formula to achieve 0 D of LCA at 500 nm. Cone absorptances as a function of wavelength were taken from the Irradiance Toolbox (Lucas et al., 2014). Although most humans are trichromats, with short (S), medium (M), and long (L) wavelength sensitive cones, as previously discussed there are several reasons why the distinction between M and L cones might not be important for emmetropization (Gawne & Norton, 2020). Therefore, we created a normalized absorptance profile (ML) by combining the profiles of the M and L cones. The profiles for the SWS and combined MLWS cones were then interpolated to the wavelengths of the hyperspectral images.

For each image, we calculated the results with the optics fixed but at different retinal positions corresponding to –3 D to +3 D in 0.25-D steps. In accordance with standard clinical practice, we used negative diopters to denote myopic defocus, because even though a myopic eye has too much optical power, it requires a negative lens to correct it.

Modeling the optics of the human eye can be quite involved, as there are many different factors, such as higher order aberrations and diffraction. However, because we were concerned with determining whether the blue cones could play a role in using chromatic cues to guide emmetropization, and because these have a spatial Nyquist sampling frequency in the range of 3 to 5 cycles per degree (cpd) (Calkins, 2001) (approximately one-tenth that of nominal human perceptual visual acuity) and higher order aberrations and diffraction have very little effect in this spatial frequency range (Artal, 2014), here we used a simplified optical model (see Discussion for more details).

**Figure 2.**
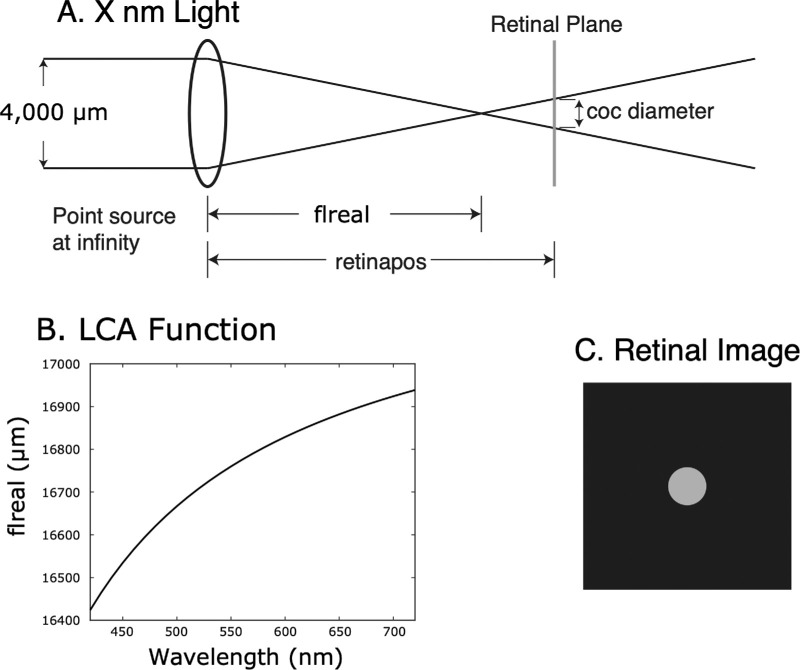
Schematic of the optical model. (A) We assume a point source at infinity, and the focal length of the lens (“flreal”) only varies with the wavelength of light. We model optical defocus blur by changing the location of the retina (“retinapos”). The image of the point of light will be a uniform disk, the circle of confusion (CoC). (B) Graph showing the mapping from the wavelength of light to focal length. (C) The retinal image of a point source of light is a uniform circular blur disk. The intensity of light within the blur disk will be proportional to the intensity of the point source divided by the area of the blur disk. Because optical systems in this regime are linear, we can then determine the retinal image of any visual scene at a specific wavelength and specific defocus by convolving the blur disk with the image of the visual scene. In essence, because a visual scene can be considered to be a superposition of a large number of points, the image on the retina is just the superposition of the corresponding blur disks.

When imaging a point of light at optical infinity, a perfect optical system that is in focus will have a physical image that is itself a single point. If the optical system is out of focus, the image will be of a relatively uniform disk: the circle of confusion, or CoC (Strasburger, Bach, & Heinrich, 2018). We calculated the diameter of this disk at a specific wavelength and defocus by first using the above formula for LCA, converting from diopters to axial distance (*dx*), and then creating a modified focal length:

flreal=16,667+dx

where *flreal* and *dx* are in µm. The diameter of the CoC is then given by

$$CoC=abs\left(\left(\left(\mathrm{flreal}-\mathrm{retinapos}\right)/\mathrm{flreal}\right)*\mathrm{pupil}\right)$$

where *retinapos* is the position of the retina (µm), and *pupil* is the diameter of the pupil (µm). Here, we always set pupil = 4000 µm. The basic optical model is illustrated in schematic form in Figure 2.

**Figure 3.**
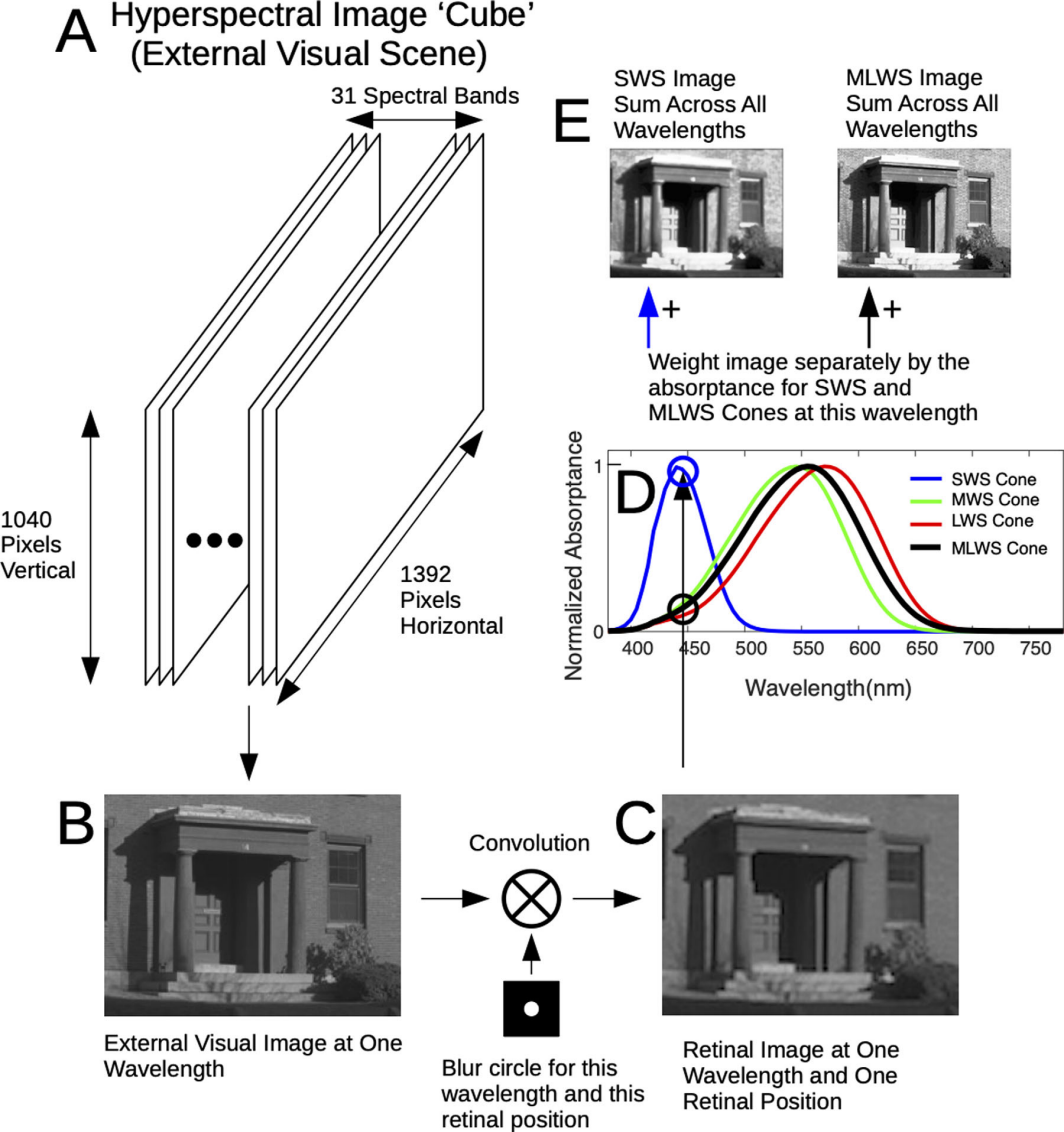
Schematic of the process by which a hyperspectral image is transformed into separate SWS and MLWS effective images. (A) The external scene is represented by a hyperspectral image “cube,” which has a single image at each of the 31 spectral bands. (B) An isolated image at a single spectral band/wavelength. (C) Using the blur circle calculated in Figure 2 for this wavelength and this retinal location, we used convolution to calculate the image on the retinal surface at this wavelength. (D) Normalized absorptance curves for the SWS cones and the MLWS cones (average of the MWS and LWS cones; see text for details). At this wavelength we determined the weighting factor for both cone types. Here, the SWS cone weighting factor was high, near 1.0, and the MLWS weighting factor was low, about 0.1. (E) Using the weighting factors, we add the image to the total summed SWS and MLWS images. The final result was two images: one representing the pattern of SWS cone activation across the retinal surface and one representing the pattern of MLWS cone activation across the retinal surface.

We then created two 401 × 401 arrays to represent the blur disks, one for the SWS cones and one for the MLWS cones. This size was chosen to ensure that the blur disks did not become larger than the arrays for the conditions used here. Initially, these arrays were set to all zeros. For each, we set a normalized blur disk, centered in the middle, with a diameter equal to CoC and an integrated intensity within the diameter of the CoC that was constant. We then calculated the two-dimensional (2D) convolution of this blur disk with the hyperspectral image at this specific wavelength. Separately for the SWS and MLWS cones, we weighted this intermediate image by the absorptance of the cones at this wavelength, multiplied by the wavelength to move from energy to relative photon counts, and then summed across all wavelengths. The result was two effective “images” (that is, 2D patterns of activity across the retinal surface): one for the SWS cones and one for the MLWS cones. We normalized each image to the same mean, as cone photoreceptors normalize their activity over a several orders of magnitude range of light intensities (Burkhardt, 1994). These effective images are what the arrays of SWS and MLWS cones would sense, given the specific hyperspectral image and specific level of defocus. This process is shown schematically in Figure 3.

We analyzed these two effective SWS and MLWS images by calculating the radial averaged 2D Fourier transform using code provided by Lawrence Sincich (Adams, Sincich, & Horton, 2007). Across all 25 images, we calculated the hyperspectral drive, which we defined as the radially averaged amplitude SWS – MLWS for each image, and we then plotted the average drive function across all 25 images as a function of spatial frequency and optical defocus.

## Results

**Figure 4.**
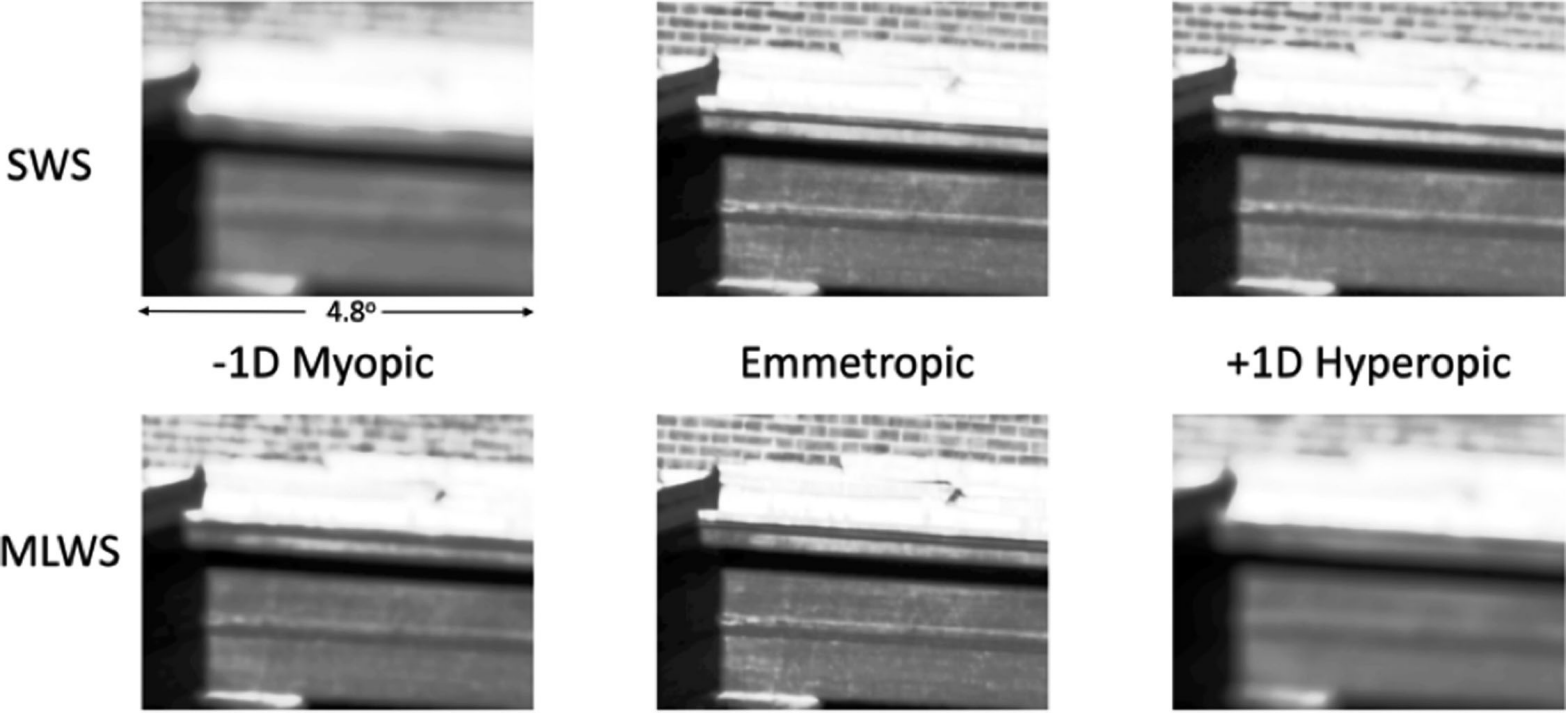
The effective images sensed by the two cone arrays as a function of defocus. As applied to a single example hyperspectral image illustrated in Figure 1 (only one-quarter of the full image is presented here), we show the effective images sensed by the array of SWS cones (top row) and the array of pooled MLWS cones (bottom row). Light indicates more photon absorption and dark less photon absorption by the cone photoreceptors. For myopic defocus (eye too long for its optics, left column) the MLWS cones “see” a relatively sharper image than the SWS cones. For hyperopic defocus (eye too short for its optics, right column) the SWS cones “see” a relatively sharper image than the MLWS cones. In accordance with standard clinical practice, we represent myopic defocus as negative diopters, as one would need a negative power lens to correct the focus.

Figure 4 illustrates an example of the calculation of the effective images as sensed by the array of SWS and MLWS cones as a function of optical defocus for a single example hyperspectral image. Because longer wavelengths focus farther back than shorter ones, for myopic defocus the MLWS cones sense a sharper image than the SWS cones, and the reverse is true for hyperopic defocus. The critical point is that, for emmetropization, the retina detects and processes two images, not one.

Unlike conscious perception, in emmetropization each patch of the retina independently integrates its visual experience over a long period of time to determine focus (Troilo et al., 2019). Whereas the fovea in humans will tend to be positioned to areas of interest in a visual scene, the rest of the retina will get an essentially random sampling of the visual world. With humans typically making three saccadic eye movements per second, that means that in 1 hour a given patch of retina will experience on the order of 10,000 random samplings of the visual environment.

**Figure 5.**
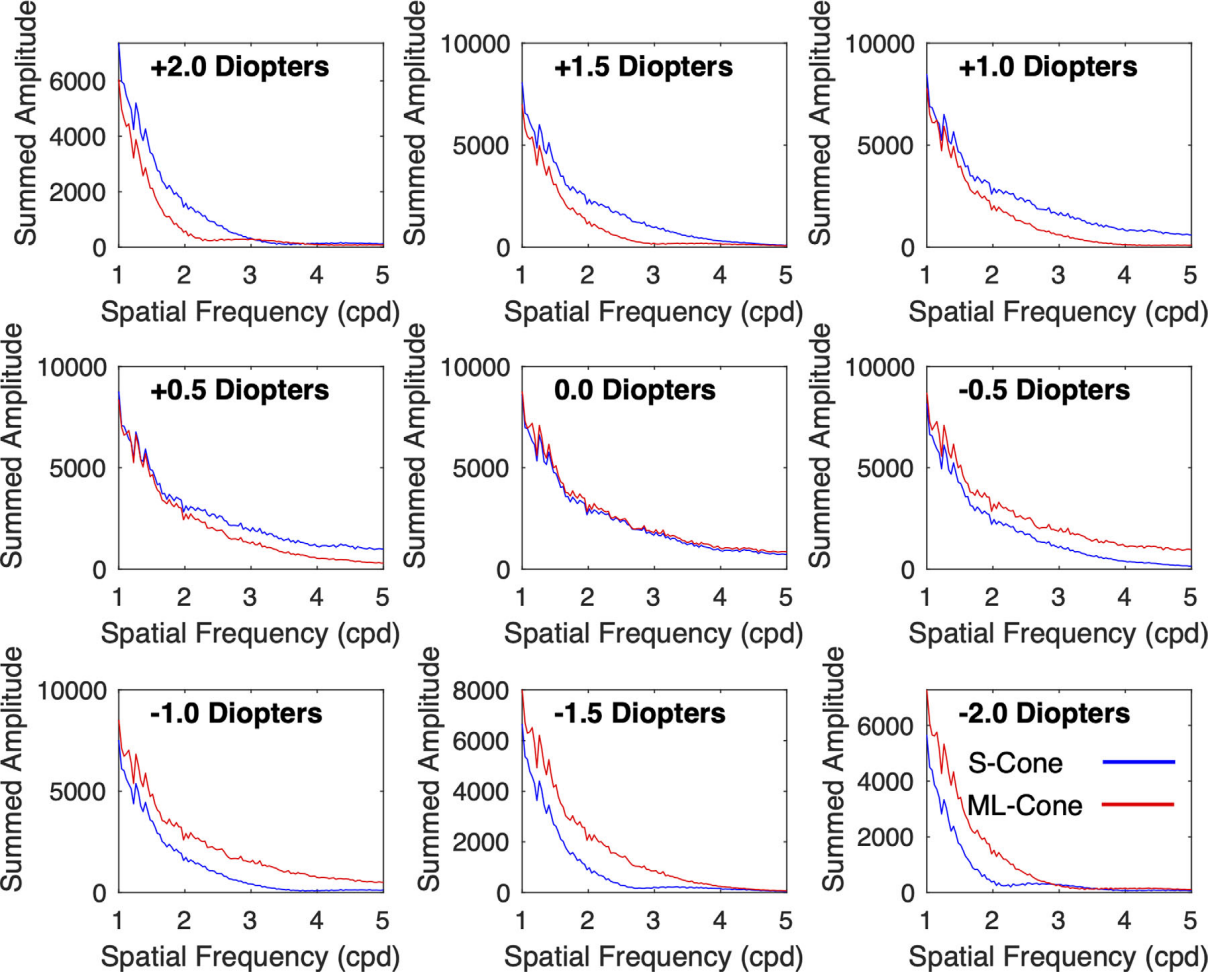
Summed signal amplitude as a function of spatial frequency and defocus for a sample image. Data were taken from the sample hyperspectral image used in Figures 1 and 4. For this analysis, we assumed that the image spanned a horizontal range of 19.1 degrees of visual angle. Positive defocus indicates hyperopia (eye too short for its optics) and minus defocus indicates myopia (eye too long for its optics).

We can simulate this process by summing the activity in the different spatial frequency bands across the entire SWS and MLWS images as a function of optical defocus. Figure 5 illustrates this for the example hyperspectral image used in Figures 1 and 4. For hyperopic defocus (positive diopters), the SWS cone array (blue lines) had significantly greater summed amplitude than the MLWS cone array (red lines) for spatial frequencies in the range of 1 to 5 cpd. In our model, this would stimulate increased axial elongation of the eye. For all levels of myopic defocus (negative diopters), the MLWS cone array (red line) had greater summed amplitude than the SWS cone array. This would produce slowed axial elongation. At emmetropia (0 D), the summed amplitude of the two arrays was approximately equal, signaling steady eye elongation that matches the maturation of the focal plane over time. These results demonstrate that differential activity between the SWS and MLWS cone arrays could decode defocus even though the SWS cone array in the peripheral human retina has an upper spatial frequency limit of around 3 to 5 cpd (Curcio, Allen, Sloan, Lerea, Hurley, Klock, & Milam, 1991).

Although a primary function of the emmetropization mechanism is to allow the resolution of high spatial frequencies in the foveal portion of the retina, the SWS cone array can plausibly provide guidance, despite its limited resolving ability, by using the lower spatial frequency information that is present when images are blurred and high spatial frequencies are not present. This is consistent with the results of studies in animals, which have found that the emmetropization mechanism does not use the high spatial frequencies, but rather the middle range of spatial frequencies to guide eyes to emmetropia (Schmid & Wildsoet, 1997; Tran, Chiu, Tian, & Wildsoet, 2008).

**Figure 6.**
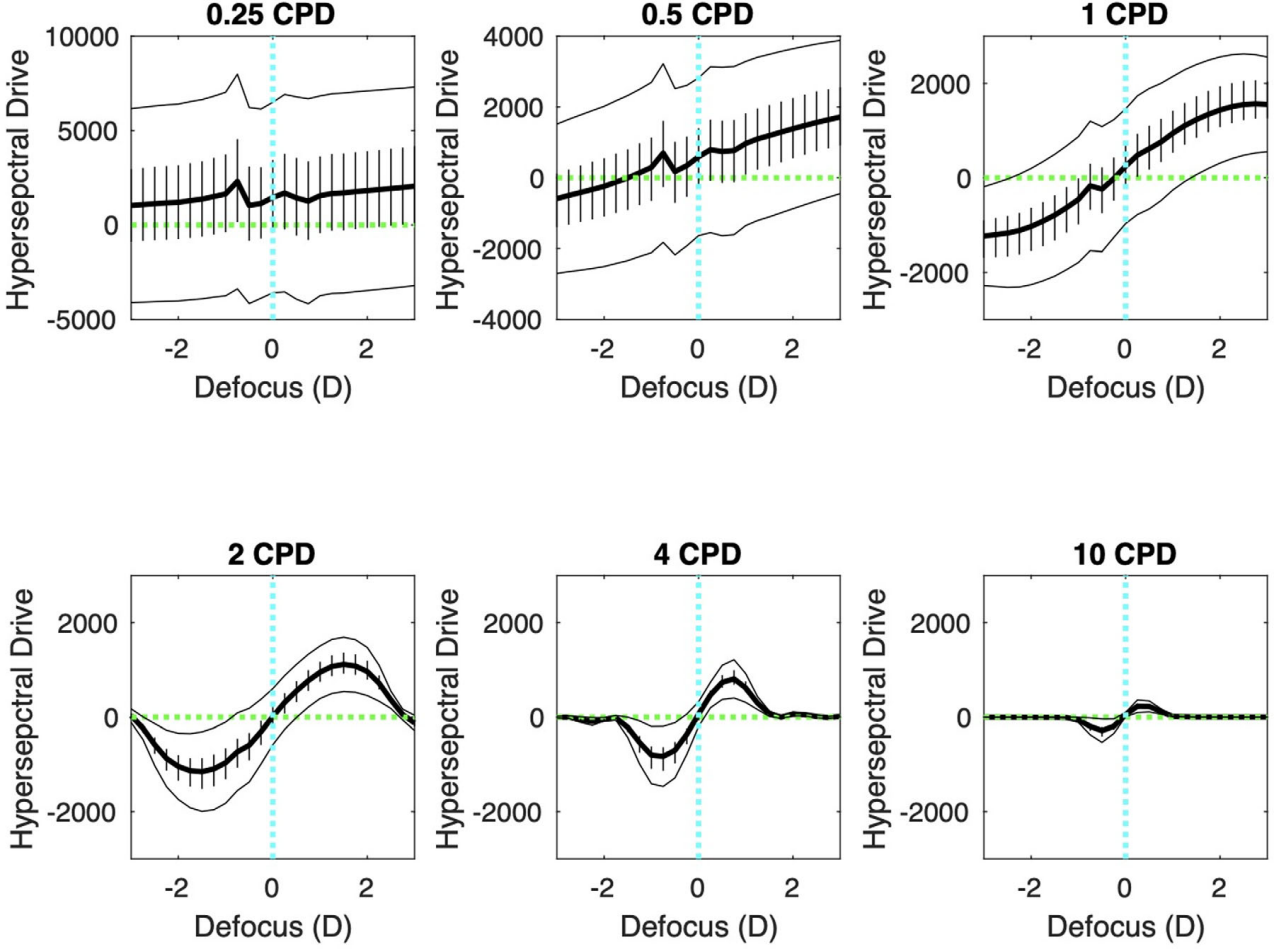
Hyperspectral drive as a function of defocus at six different spatial frequencies. The heavy black line is the mean hyperspectral drive (SWS – MLWS) of 25 real-world hyperspectral images, the error bars are the bootstrapped 95% confidence intervals, and the thin black lines are the mean ± standard deviation. For myopic (negative) defocus, the hyperspectral drive was on average negative, producing slowed axial elongation. For hyperopic (positive) defocus, the hyperspectral drive was on average positive, stimulating increased axial elongation.

We have defined the hyperspectral drive as the difference between the summed response of the SWS cone array and the MLWS cone array: SWS – MLWS. Figure 6 illustrates the hyperspectral drive as a function of image defocus for six representative spatial frequencies, averaged across 25 real-world hyperspectral images. At 0.25 and 0.5 cpd, the drive was highly variable and not very accurate (drive not significantly different from 0 across a broad range of defocus). At 1 cpd, the drive function was less variable across scenes and on average more accurate (although still quite variable between scenes). At 2 cpd, the drive was even less variable and still accurate but lost effectiveness beyond about ±2 D of defocus. At 10 cpd, the effective range of the drive function shrank to less than ±1 D.

Although the magnitude of LCA in diopters is not affected by changes in pupil size, the diameter of the CoC will change in direct proportion to the pupil size. Therefore, we repeated this analysis using pupil diameters of 2 mm and 6 mm. It should be noted that because of the Stiles–Crawford effect, for cone vision the sensitivity to light falls off dramatically as pupil size extends past a certain diameter (Stiles & Crawford, 1933). Going from a 7-mm to an 8-mm-diameter pupil, the increase in area is about 30%, but the effective increase in light-gathering capacity is only about 8%.

Supplementary Figure S2 shows the results for a pupil diameter of 2 mm, and Supplementary Figure S3 for a pupil diameter of 6 mm. The same basic pattern exists as for a pupil diameter of 4 mm, but for the smaller pupil the absolute magnitude of the hyperspectral drive was less, although it spanned a greater range of defocus. For the larger pupil diameter, the magnitude of the hyperspectral drive was increased, although the range of defocus over which it can operate was reduced. Clearly, changes in pupil size have the potential to affect the use of LCA to guide emmetropization, but, at least for the images used here, the overall pattern is not altered.

## Discussion

Although we have previously hypothesized that chromatic cues are fundamental for emmetropization (Gawne et al., 2018), this mechanism likely uses multiple visual cues (Norton, Khanal, & Gawne, 2021) and, even for chromatic ones, likely integrates them across some range of spatial frequencies. However, this analysis suggests that there is a “sweet spot” for the use of chromatic signals in emmetropization very roughly in the range of 1 to 3 cpd, within the resolution of the widely spaced SWS cones, estimated to have a Nyquist frequency of approximately 4 cpd at an eccentricity of 10° (Calkins, 2001). We note that, for a variety of animal models, emmetropization can detect the sign of defocus over a range of at least several diopters (Troilo et al., 2019). This surely makes sense, as otherwise how could emmetropization correct for poor focus? We cannot rule out that the higher spatial frequencies play a role in emmetropization, but this analysis indicates that the higher spatial frequencies are neither strictly necessary nor by themselves sufficient, in addition to not being readily detectable by the S cones.

We could have assumed a grayscale world with a spectrally flat broadband illuminant, as we did in a previous paper (Gawne & Norton, 2020). That would have shown that emmetropization could use chromatic cues given existing blue cone spacing under ideal conditions but still would not have demonstrated that it could have functioned in the real world, which was the point of this study. The possibility that the chromatic structure of some classes of visual scenes—perhaps under artificial illuminants or the restricted bandwidth of video displays—could reduce the effectiveness of emmetropization is a possibility that should be further explored. We also note that here we modeled the optics of adults, but human children have significantly greater amounts of LCA (Wang, Candy, Teel, & Jacobs, 2008). Most human children initially emmetropize normally and only become myopic later on life (Gwiazda, Thorn, Bauer, & Held, 1993). The possibility that the increased susceptibility of children to the development of myopia with age could be related to decreased LCA is another factor that should be explored.

We used simple geometric optics, but real eyes have many sources of blur besides defocus, including diffraction and higher order aberrations. However, these sources of blur typically have a negligible impact on the spatial frequencies which the blue cone mosaic can resolve (Artal, 2014), so it seems very unlikely that they play a significant role in emmetropization, except perhaps in pathological conditions. However, in addition to LCA, there is also transverse chromatic aberration (TCA), where the short- and long-wavelength retinal images are radially displaced on the retina. Unlike LCA, TCA is idiosyncratic among individuals (at least in humans) and varies in magnitude across the surface of the retina, making it less likely than LCA to be used as a cue for emmetropization. However, by reducing the spatial registration of short- and long-wavelength images on the retina, TCA could potentially interfere with the ability of the retina to compare the image contrast at short and long wavelengths of light. Although retinal neural processing is local, we do not know the integration “patch size” that the retina uses to compare short- and long-wavelength image statistics (assuming, of course, that it is in fact doing that). Nevertheless, as TCA is typically only about 6 arc minutes at an eccentricity of 20° (Thibos, Bradley, Still, Zhang, & Howarth, 1990), TCA does not appear to be an insurmountable obstacle for emmetropization to use chromatic cues as we have outlined here, although the extreme peripheral chromatic distortions of high-index refraction spectacle lenses could potentially be an issue.

Like emmetropization, accommodation must sense the magnitude and sign of blur, and there are parallels between them (although this may be due to their both having to work within the same optical constraints and does not prove the existence of a shared mechanism). The optimal spatial frequency in humans for accommodation is between 3 and 5 cpd (Mathews & Kruger, 1994; Owens, 1980). In principal, chromatic signals for blur are much more powerful than monochromatic signals (Burge & Geisler, 2011), and, in practice, human dynamic accommodation is to a great extent driven by chromatic cues (Aggarwala, Nowbotsing, & Kruger, 1995; Cholewiak, Love, & Banks, 2018).

As we have demonstrated here for humans, the sparse but regular array of SWS cones, in conjunction with the MLWS cone array, is in principle sufficient for LCA to detect the sign and the amount of defocus and provide input to the emmetropization mechanism to guide eye growth to emmetropia. In dichromatic tree shrews, we also have found that the S cone array also can participate with an L cone array in using LCA to guide emmetropization (Gawne & Norton, 2020). Many mammals have a similar pattern where the SWS cones are comparatively sparse but also relatively uniformly spread across the retinal surface (Hunt & Peichl, 2014). The retinal spacing of SWS cones may therefore be an evolutionarily ancient adaption in the mammalian line that allows the efficient use of chromatic cues in emmetropization. Even in the mouse, although it has a strong dorsal–ventral gradient of S-cone opsin, the distribution of “true” S cones connected to specific S-cone bipolar cells also seems to follow this pattern; it has been suggested that this is the primordial color system of the mammalian retina (Haverkamp, Wassle, Duebel, Kuner, Augustine, Feng, & Euler, 2005). In contrast, the LWS (in dichromatic species) and MLWS cones have evolved much denser cone spacings that, in addition to their role in emmetropization, also mediate greater perceptual visual acuity, although this greater acuity could only be utilized if emmetropization using both types of cones had achieved and maintained well-focused images on the retina during postnatal development.

## Supplementary Material

Supplement 1

Supplement 2

Supplement 3
