# Supplementary material for: How chromatic cues can guide human eye growth to achieve good focus
Source: J Vis. 2021 May 13;21(5):11. doi: 10.1167/jov.21.5.11 (PMC8131997; doi:10.1167/jov.21.5.11)
Supplement: Supplement 1 [file jovi-21-5-11_s001.pdf]

Supplementary Figure 1.

Subset of the hyperspectral images taken from Chakrabarty, A., & Zickler, T. (2011, 2011). *Statistics of Real-World Hyperspectral Images.* Paper presented at the Proc. of the IEEE Computer Science Society Conference on Computer Vision and pattern Recognition. Shown are synthetized red-green-blue images with the colors hisogram equalized.

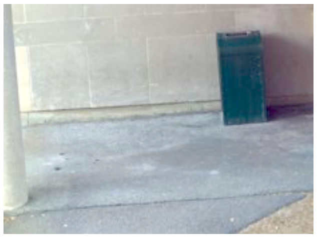
1. imga7 sparse urban concrete

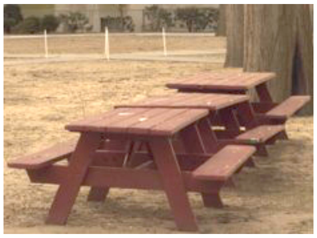
2. imgb0 outdoor rectangular picnic tables

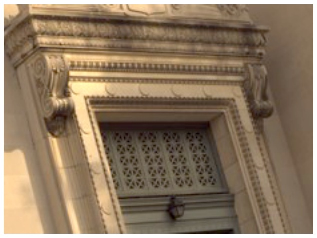
3. imgb5 outdoor rectangular arch a lot of fine rectilinear detail

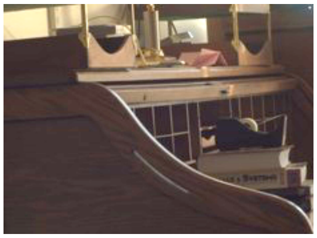
4. imgd2 inside daylight old fashioned roller desk

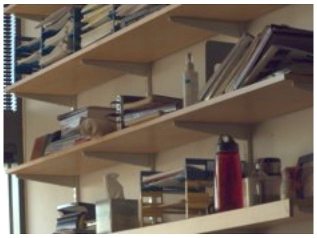
5. imgd3 daylight inside shelves with books and stuff

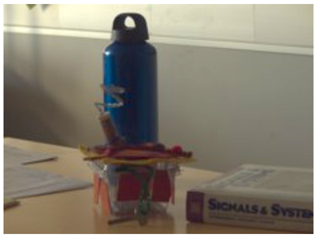
6. imgd4 daylight inside some weird thing

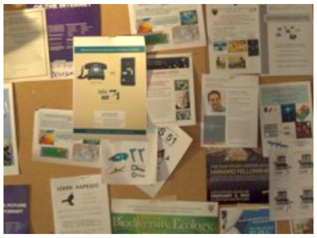
7. imgd7 daylight inside papers on a bulletin board

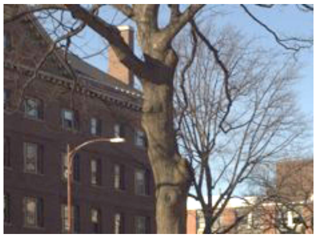
8. imgf2 outdoor tree against sky and building

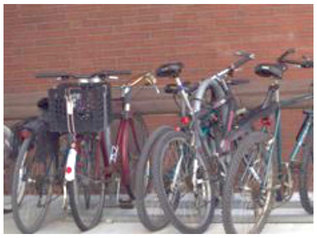
9. imgf5 outside bicycles lined up

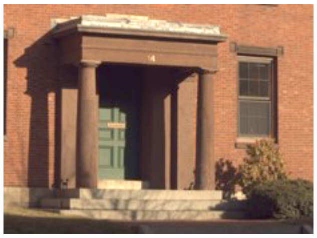
10. imgf7 outside covered entrance brick building EXEMPLAR IMAGE

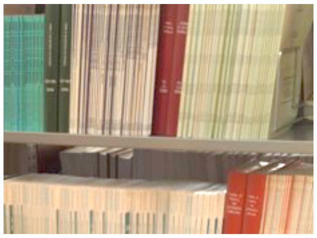
11. imgh0 inside daylight ends of a bunch of magazines

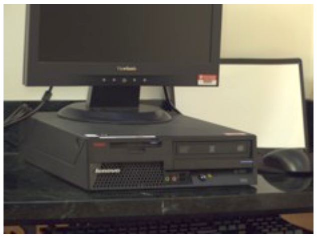
12. imgh1 inside daylight computer on desk

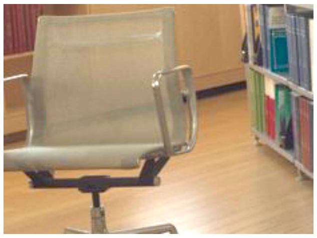
13. imgh3 inside daylight office chair

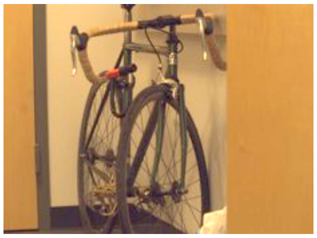
14. img4 single bicycle in hall artificial lighting

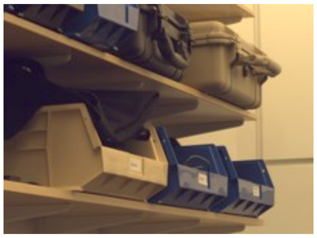
15. img6 plastic bins on a shelf artificial lighting

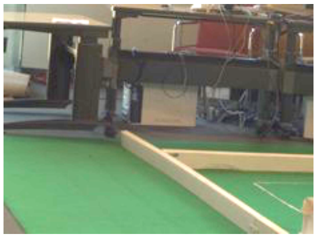
16. imga4 artificial lighting benches and stuff

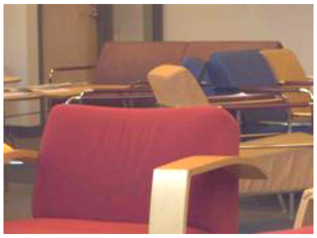
17. imga8 artificial lighting chairs

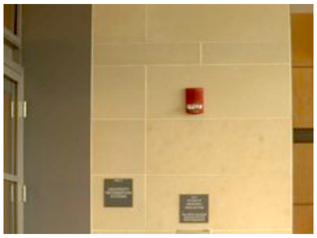
18. imgc3 artificial light relatively blank tile wall

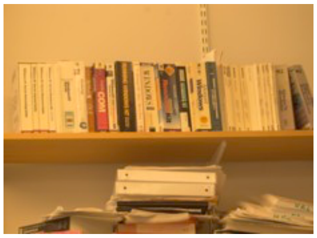
19. imgd0 artificial light books on a bookshelf

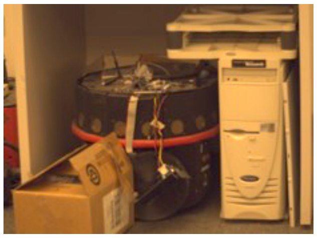
20. imgd1 artificial light computer and stuff

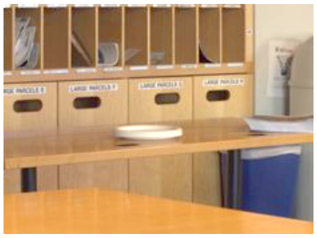
21. imgd5 artificial light bins and shelves

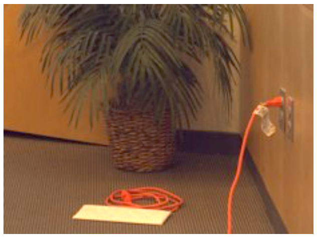
22. imgd6 artificial light electric plug near potted plant

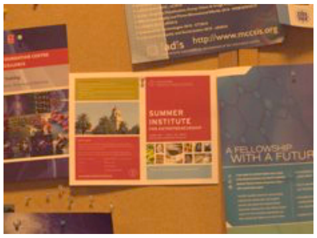

23. imgg0 artificial light notices stuck on a bulletin board

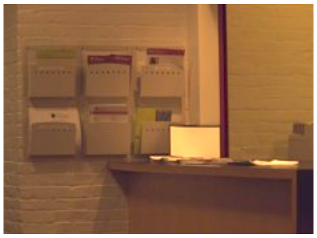
24. imgg2 artificial light random office scene

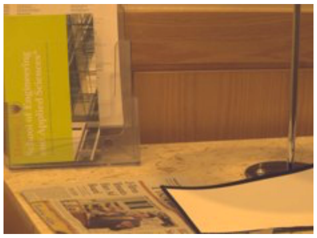
25. imgg5 artificial light papers desk etc.
