# Supplementary figures and images for: How chromatic cues can guide human eye growth to achieve good focus
Source: J Vis. 2021 May 13;21(5):11. doi: 10.1167/jov.21.5.11 (PMC8131997; doi:10.1167/jov.21.5.11)

plot hyperspectral frequency 2mmpupil

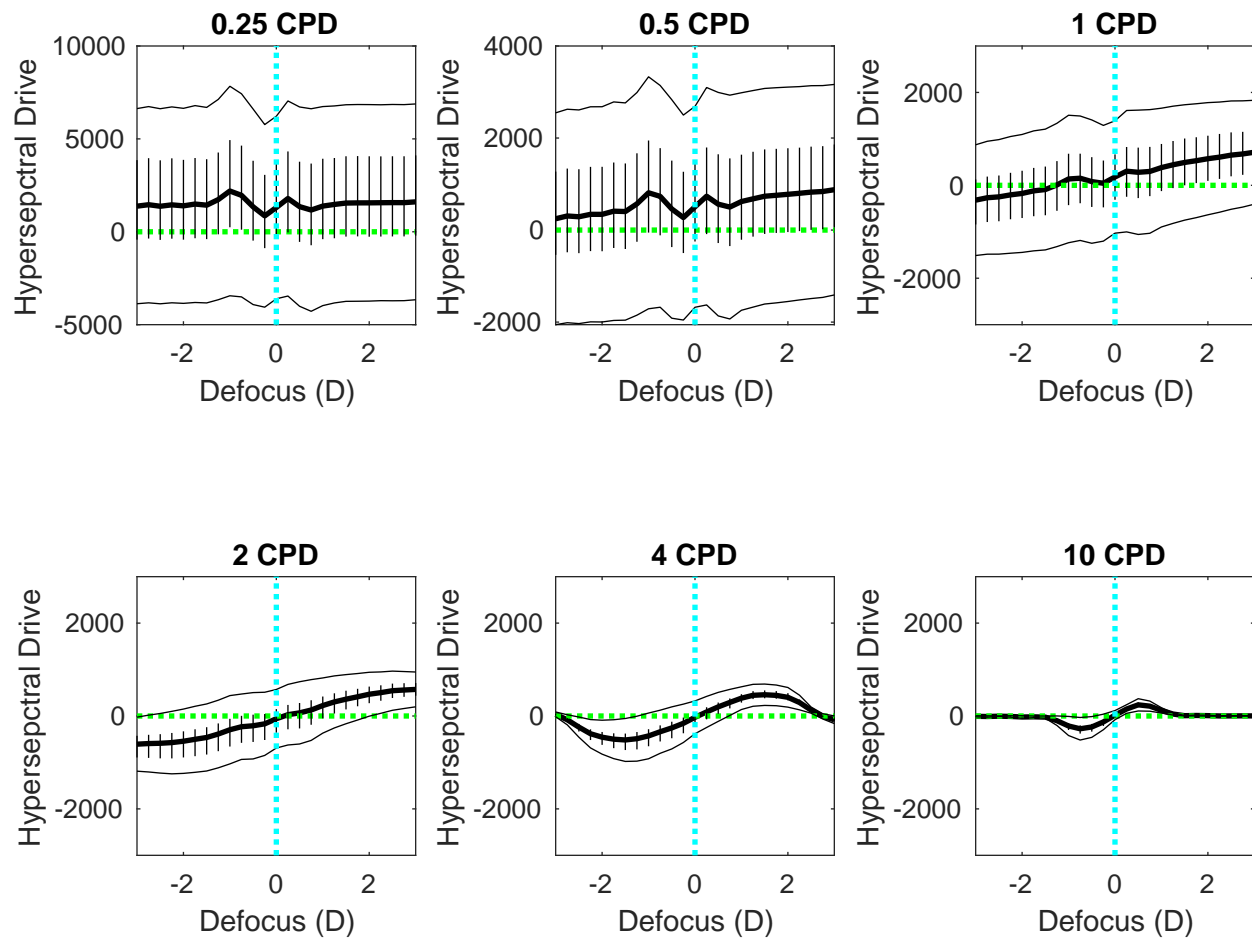

Supplement: Supplement 2 [file jovi-21-5-11_s002.pdf]

plot hyperspectral frequency 6mmpupil

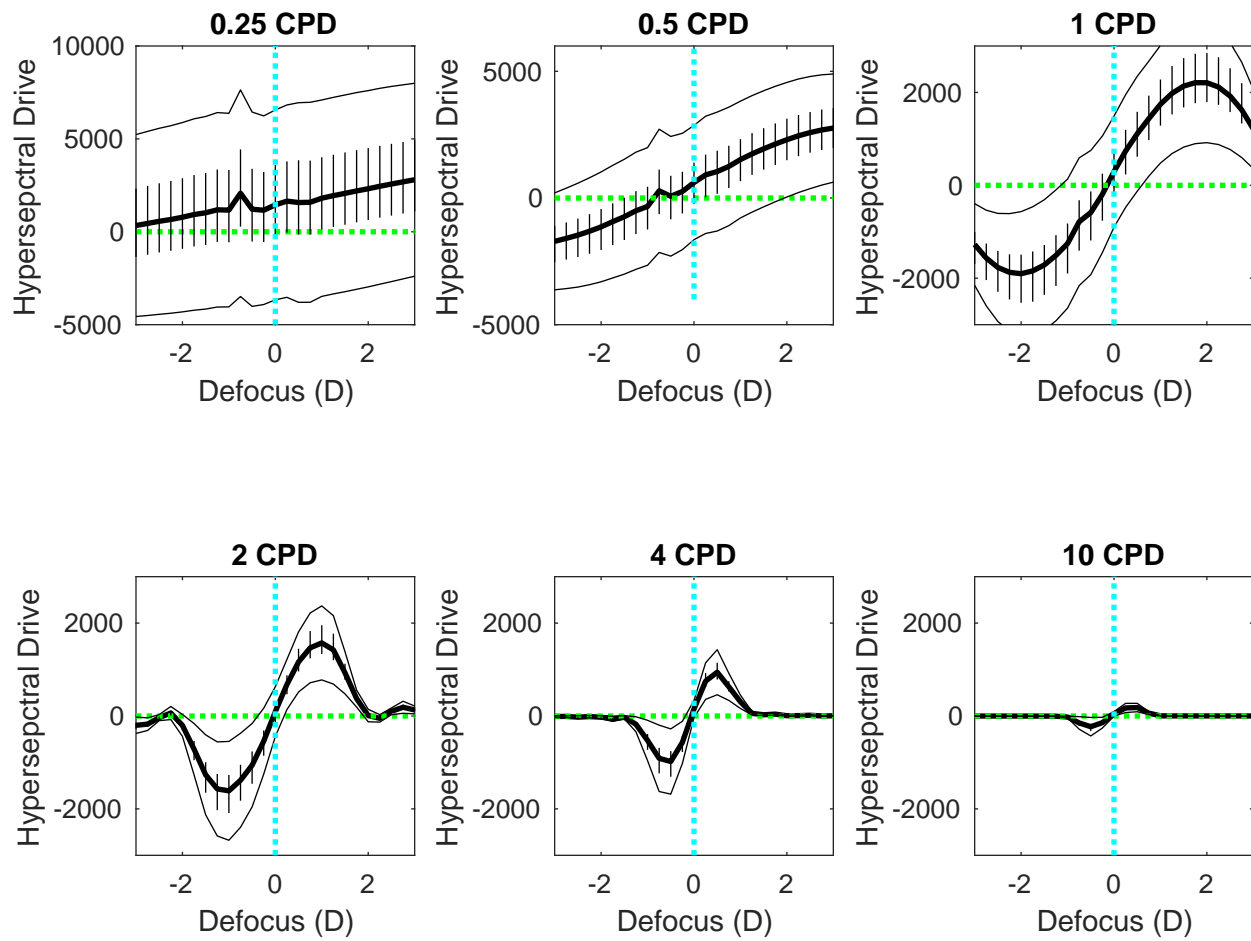

Supplement: Supplement 3 [file jovi-21-5-11_s003.pdf]
